# An updated checklist of the genus *Rubus* (Rosaceae), a proposal of a new name, *Rubus subbeskidensis*, and an analysis of micro-hotspots of bramble diversity in Poland

**DOI:** 10.3897/phytokeys.277.196859

**Published:** 2026-07-16

**Authors:** Marcin Nobis, Mateusz Wolanin, Adam Zając, Bohumil Trávníček

**Affiliations:** 1 Institute of Botany, Faculty of Biology, Jagiellonian University, Gronostajowa 3, 30-387 Kraków, Poland Institute of Botany, Faculty of Biology, Jagiellonian University Kraków Poland; 2 Faculty of Biology, Nature Protection and Sustainable Development, Rzeszów University, Zelwerowicza 4, 35-601 Rzeszów, Poland Faculty of Biology, Nature Protection and Sustainable Development, Rzeszów University Rzeszów Poland; 3 Plant Biosystematics and Ecology Research Group, Department of Botany, Faculty of Science, Palacký University, Šlechtitelů 27, 77900 Olomouc, Czech Republic Plant Biosystematics and Ecology Research Group, Department of Botany, Faculty of Science, Palacký University Olomouc Czech Republic

**Keywords:** Diversity, distribution, micro-hotspots, phytogeography, Poland, *
Rubus
*, taxonomy

## Abstract

The genus *Rubus* is among the most taxonomically complex and species-rich groups of vascular plants in Europe, owing to extensive morphological variation, polyploidy, facultative apomixis and frequent hybridisation. An updated checklist of the genus *Rubus* in Poland, presented here, comprises 113 taxa, including 106 native species and seven established alien taxa; 12 species occur exclusively in Poland and are treated as regional endemics. An illegitimate later homonym, *Rubus magurensis*, is replaced here with the new name, *Rubus subbeskidensis*. Using 88,934 verified records from the ATPOL database, spatial patterns of species richness are analysed and seven regional micro-hotspots of *Rubus* diversity in Poland are identified. The highest concentrations of species are located in south-western Poland, particularly in the Kotlina Kłodzka Basin, the Moravian Gate and the forelands of the northern Carpathians. Phytogeographical analyses of the distribution patterns of all brambles occurring in Poland allow the recognition of four distinct geographical elements, comprising species reaching their northern, north-eastern, western and southern distribution limits in Poland. The distribution patterns of selected species groups depend mainly on specific bioclimatic factors determining the occurrence of individual species.

## Introduction

The genus *Rubus* comprises over 750 species in Europe. However, owing to high morphological diversity, polyploidy, facultative apomixis and frequent hybridisation, members of the genus are among the most taxonomically challenging groups of vascular plants (Kollmann et al. 2000; Kurtto et al. 2010; Sochor et al. 2025). The taxonomy of the genus is further complicated by the presence of microspecies in apomictic complexes and ongoing processes of speciation, not to mention the existence of a number of temporally local biotypes of little or no taxonomic significance (Sochor et al. 2025).

Following Kurtto et al. (2010), only a few diploid and sexual species can be treated as true biological species in the modern taxonomy of *Rubus*, while all other brambles form an apogamous complex of biotypes more or less stabilised by apomixis. Stabilised biotypes distinguished by the constancy of morphological characters and ecological demands, with an appreciable distribution area, are easy to recognise and are treated as species. In Europe, it is accepted that only widely distributed biotypes (geographical range diameter 500–1000 km), regional biotypes with some tendency to a wide distribution (250–500 km), regional biotypes (50–250 km) and, exceptionally, local biotypes with some tendency to regional distribution (20–50 km) should be treated as species, thus excluding local or singular biotypes with a geographical range diameter of less than 20 km. However, it is worth noting that the number of local biotypes of brambles in Europe is extremely high (Sochor et al. 2025), and thus the taxonomy of *Rubus* and species richness in a given area still require advanced studies.

Twenty years ago, Zieliński (2004), in a monograph dedicated to the genus *Rubus*, listed and characterised in detail 88 species occurring in Poland. Since then, a number of species have been described as new to science or newly recorded in Poland (Trávníček and Zázvorka 2005; Trávniček et al. 2005; Kosiński 2006, 2010; Kosiński and Oklejewicz 2006; Kosiński and Zieliński 2013; Oklejewicz et al. 2013; Kosiński et al. 2014, 2018; Maliński et al. 2014, 2015; Wolanin et al. 2016, 2020). In the recent checklist of vascular plants in Poland (Mirek et al. 2020), 109 species of brambles are accepted, while the distribution maps of nearly all *Rubus* species in Poland were presented by Zając and Zając (2001) and Zieliński (2004). Several additional maps, updated on a relatively regular basis, have been published by Zając and Zając (2019). Nevertheless, phytogeographical analyses of the distribution of these species in Poland have not been the subject of separate studies. The first attempt to present a map of bramble species richness in Poland was undertaken by Zieliński (2004). The author emphasised that south-western Poland constitutes a centre of *Rubus* diversity, with more than 70 species recorded there (out of 88 species known from the country at that time), whereas only five species occurred in north-eastern regions. This high species richness observed in south-western Poland (> 26 species per 10 × 10 km cartogram unit) was explained by the author as being determined primarily by climatic conditions and dispersal pathways of brambles (Zieliński 2004). It should be noted, however, that only after the publication of Zieliński (2004) did a number of floristic papers appear, reporting many localities of species previously unknown from these areas (e.g. Nobis 2007; Piwowarczyk 2008; Nobis 2008; Wolanin 2013; Nykiel et al. 2014; Pierścińska 2014; Jaźwa and Stadnicka-Futoma 2017), as well as studies explicitly dedicated to the taxonomy, ecology and distribution of brambles in various regions of Poland (e.g. Maliński 2001; Zieliński et al. 2004; Oklejewicz 2006; Kosiński 2010; Gapińska and Kosiński 2016).

Since the genus *Rubus* is among the most species-rich genera in Poland, in which almost every year brings new records or new species described from this area, the aim of this paper is to present an updated list of brambles in Poland and provide an analysis of micro-hotspots of bramble species diversity in Poland, as well as a phytogeographical analysis of species distributions, the so-called analysis of geographical elements of *Rubus* in the flora of Poland. Additionally, *R. magurensis* Wolanin, M. Nobis & Oklej. (Wolanin et al. 2025) was found to be an illegitimate name; therefore, a new species name, *R. subbeskidensis*, is proposed for this taxon.

## Materials and methods

The checklist of Polish brambles, including one new name proposed for the recently published *Rubus magurensis* nom. illeg., has been updated and compiled on the basis of relevant taxonomic literature and a revision of herbarium specimens deposited mainly in KRA, KRAM, KOR, POZ and GDA (acronyms given according to Thiers 2025), and, where available, their digitised images online. Species whose occurrence was confirmed before 2003 were listed and taxonomically and morphologically characterised in detail by Zieliński (2004). For every other species whose occurrence in Poland was confirmed in or after 2004, information is provided on the author(s) of the first record, together with notes regarding the finding and, if necessary, taxonomic notes. Brambles erroneously reported from Poland, whose occurrence was not confirmed by herbarium specimen(s) or field studies, as well as the names of published local biotypes, were omitted. This information can be found in full in Mirek et al. (2020). Since local biotypes have minor or no taxonomic value and are usually omitted from national species lists, it was decided not to provide these names here; however, all of them can be the subject of further taxonomic studies, especially during revisions focused on selected series or taxonomically complicated species groups, using integrated morpho-molecular methods. A long list of synonyms of accepted *Rubus* names already published by Zieliński (2004) and Mirek et al. (2020) was also omitted, with the exception of a few taxa for which new taxonomic and nomenclatural information has been published recently.

The analysis of the distribution of all representatives of brambles in Poland was conducted on the basis of data deposited in the ATPOL database (Zając 1978), which includes 88,934 records of *Rubus* species in Poland occurring in 3,157 basic cartogram units of 10 × 10 km (ATPOL 2025). Because most of the brambles occurring in Poland reach the species-specific limits of their geographical range, four species-specific range groups of *Rubus*, corresponding to specific geographical elements in the flora of Poland (Zając and Zając 2009), were distinguished based on detailed analyses of distribution maps. The maps of *Rubus* species richness and geographical elements of brambles in Poland were prepared and analysed in the ATPOL database.

## Results and discussion

### Updated list of *Rubus* species occurring in Poland

The list of *Rubus* taxa is presented in alphabetical order. Names given in bold are currently accepted. Explanations of symbols used in the list: *. Polish names of accepted species are given in brackets. For each species whose occurrence in Poland was confirmed in or after 2004 and that was not included in the monograph of Zieliński (2004), information on the first record of the species in Poland is provided after the dash, with notes regarding the finding and, where necessary, taxonomy.

***Rubus acanthodes*** (H. Hofm. ex Focke) E. Barber (jeżyna saksońska)

*Rubus allegheniensis Porter (jeżyna alegeńska)

*Rubus ambrosius* Trávn. & Oklej. = **Rubus canaliculatus P.J. Müll**.

***Rubus angustipaniculatus*** Holub (jeżyna rombolistna)

***Rubus apricus*** Wimm. (jeżyna słoneczna)

***Rubus × areschoughii*** A. Blytt (jeżyna Areschougha) – this taxon is of a hybrid origin between *R. caesius* and *R. saxatilis*

*Rubus armeniacus Focke (jeżyna kaukaska)

***Rubus atrovirens*** P.J. Müll. (jeżyna ciemnozielona)

***Rubus austroslovacus*** Trávn. (jeżyna słowacka) – the first record from Poland was published by Trávníček and Zázvorka (2005), and the distribution map of the species in Poland was later supplemented by Zieliński et al. (2004b), Kosiński (2010) and Oklejewicz in Zając and Zając (2019)

***Rubus barberi*** H.E. Weber (jeżyna Barbera)

***Rubus bavaricus*** (Focke) Hruby (jeżyna bawarska)

***Rubus bicolor*** Opiz (jeżyna dwukolorowa) – earlier, this taxon was named *R. montanus* auct. mult. non Lib. ex Lej. (Király et al. 2017); the distribution map of this species under the name *R. montanus* Lib. ex Lej. in Poland was published by Zając and Zając (2019)

***Rubus bifrons*** Vest (jeżyna dwubarwna)

***Rubus bohemo-polonicus*** Trávn. & Ziel. (jeżyna pograniczna) – the first record from Poland was published by Zieliński and Trávníček (2004)

***Rubus caesius*** L. (jeżyna popielica)

***Rubus camptostachys*** G. Braun (jeżyna orzęsiona)

*Rubus canadensis L. (jeżyna kanadyjska)

***Rubus canaliculatus*** P.J. Müll. (jeżyna wzniesiona) – the first record from Poland was published under the name *Rubus ambrosius* by Trávníček et al. (2005); however, the latter was recently treated as conspecific with *R. canaliculatus* by Hassler and Muer (2024)

***Rubus capitulatus*** Utsch (jeżyna główkowata)

***Rubus capricollensis*** (Sprib.) Sprib. (jeżyna krótkopręcikowa)

***Rubus chaerophylloides*** Sprib. (jeżyna świerząbkolistna)

***Rubus chaerophyllus*** Sagorski & Wilh. Schulze (jeżyna świerząbkowata)

***Rubus chamaemorus*** L. (malina moroszka)

***Rubus chlorothyrsos*** Focke (jeżyna wielolistna)

***Rubus circipanicus*** E.H.L. Krause (jeżyna meklemburska)

***Rubus clusii*** Borbás (jeżyna Kluzjusza) – the first record from Poland was published by Oklejewicz et al. (2013), and its distribution map was supplemented by Oklejewicz and Chwastek (2017)

***Rubus constrictus*** P.J. Müll. & Lefèvre (jeżyna Westa)

*Rubus corylifolius* Sm. agg. – this group includes many taxa and local biotypes classified as *Rubus* sect. *Corylifolii*; Mirek et al. (2020) treated it in Poland as a collective species; the group requires revision using methods of integrative taxonomy

***Rubus crispomarginatus*** Holub (jeżyna kędzierzawolistna)

***Rubus curvaciculatus*** Walsemann ex H.E. Weber (jeżyna drobnokolczasta)

***Rubus czarnunensis*** (Sprib.) Sprib. (jeżyna notecka)

***Rubus divaricatus*** P.J. Müll. (jeżyna połyskująca)

***Rubus divulgatus*** Sochor (jeżyna rozpowszechniona) – the first record from Poland, together with detailed morphological characters and taxonomy, was published by Sochor et al. (2025)

***Rubus dollnensis*** Sprib. (jeżyna dolnośląska)

***Rubus fabrimontanus*** (Sprib.) Sprib. (jeżyna podgórska)

***Rubus fasciculatus*** P.J. Müll. (jeżyna szarozielona)

*Rubus flos-amygdali* Trávn. & Holub = **Rubus montanus** Lib. ex Lej.

***Rubus franconicus*** H.E. Weber (jeżyna frankońska)

***Rubus glandulosus*** Bellardi (jeżyna gruczołowata)

***Rubus glivicensis*** (Sprib. ex Sudre) Sprib. (jeżyna gliwicka)

***Rubus gothicus*** Frid. & Gelert ex E.H.L. Krause (jeżyna gocka)

***Rubus grabowskii*** Weihe (jeżyna bukietowa)

***Rubus gracilis*** J. Presl & C. Presl (jeżyna ostręga)

***Rubus graecensis*** W. Maurer (jeżyna austriacka)

***Rubus gratus*** Focke (jeżyna nadobna)

***Rubus guentheri*** Weihe (jeżyna Günthera)

***Rubus guttiferus*** Trávn. & Holub (jeżyna kropelkowa) – the first record from Poland, together with detailed morphological characters and taxonomy, was published by Trávníček and Zázvorka (2005); the distribution map of the species in Poland was supplemented by Kosiński (2006, 2010) and presented by Zając and Zając (2019)

***Rubus henrici-egonis*** Holub (jeżyna Webera)

***Rubus hercynicus*** G. Braun (jeżyna hercyńska)

***Rubus hevellicus*** (E.H.L. Krause) E.H.L. Krause (jeżyna Aschersona)

*Rubus hirtus* Waldst. & Kit. = **Rubus glandulosus** Bellardi

***Rubus holzfussii*** Sprib. (jeżyna Holzfussa)

***Rubus × idaeoides*** Ruthe (jeżyna malinowa) – this taxon is of hybrid origin between *R. caesius* and *R. idaeus*

***Rubus idaeus*** L. (malina właściwa)

***Rubus kaznowskii*** Ziel. & Kosiński (jeżyna Kaznowskiego) – detailed morphological characters, the taxonomy of this species newly described from Poland and its distribution map were published by Kosiński et al. (2021)

***Rubus koehleri*** Weihe (jeżyna Köhlera)

***Rubus kuleszae*** Ziel. (jeżyna Kuleszy)

*Rubus laciniatus Willd. (jeżyna wcinanolistna)

***Rubus lamprocaulos*** G. Braun (jeżyna skąpokwiatowa)

***Rubus lidforsii*** (Gelert) Lange (jeżyna Lidforsa)

***Rubus lignicensis*** Figert (jeżyna legnicka)

***Rubus limitaneus*** Maliński & Ziel. (jeżyna pomorska) – detailed morphological characters and taxonomy of this species, newly described from Poland, were published by Maliński et al. (2014)

***Rubus lindebergii*** P.J. Müll. (jeżyna Lindeberga) – the first record of this species from Poland was published by Maliński et al. (2015)

***Rubus lucentifolius*** Ziel. & Kosiński (jeżyna połyskliwolistna) – detailed morphological characters and taxonomy of this species, newly described from Poland, were published by Zieliński et al. (2004a)

***Rubus lusaticus*** Rostock (jeżyna łużycka)

***Rubus macrophyllus*** Weihe & Nees (jeżyna wielkolistna)

*Rubus magurensis* Wolanin, M. Nobis & Oklej., nom. illeg. = **Rubus subbeskidensis** Wolanin, M. Nobis & Oklej.

***Rubus marssonianus*** H.E. Weber (jeżyna Marssona)

***Rubus maximus*** T. Marsson (jeżyna większa) – the first record of this species from Poland was published by Kosiński and Zieliński (2013)

***Rubus micans*** Godr. (jeżyna lśniąca)

***Rubus mollis*** J. Presl & C. Presl (jeżyna szarolistna)

***Rubus montanus*** Lib. ex Lej. (jeżyna wąskolistna) – earlier, this taxon was named *R. flos-amygdali* Trávn. & Holub (Király et al. 2017); the distribution map of this species in Poland, under the name *R. flos-amygdali*, was published by Zając and Zając (2019)

***Rubus nemoralis*** P.J. Müll. (jeżyna smukłokolcowa)

***Rubus nemorosus*** Hayne & Willd. (jeżyna zaroślowa)

***Rubus nessensis*** Hall (jeżyna lochneseńska)

***Rubus nigricans*** Danthoine (jeżyna czarniawa)

***Rubus oboranus*** (Sprib.) Sprib. (jeżyna trójlistkowa)

*Rubus occidentalis L. (jeżyna zachodnia) – the first record of this species from Poland was published by Kosiński et al. (2014)

*Rubus odoratus L. (jeżyna pachnąca)

***Rubus oklejewiczii*** Wolanin & M. Nobis (jeżyna Oklejewicza) – detailed morphological characters and taxonomy of this species, newly described from Poland, were published by Wolanin et al. (2020)

***Rubus opacus*** Focke (jeżyna ponura)

***Rubus orthostachys*** G. Braun (jeżyna prostokwiatostanowa)

***Rubus ostroviensis*** Sprib. (jeżyna ostrowska)

***Rubus pallidus*** Weihe (jeżyna bladozielona)

***Rubus parthenocissus*** Trávn. & Holub (jeżyna winobluszczowa) – the first record of this species from Poland was published by Trávníček and Zázvorka (2005), and its distribution was supplemented by Kosiński and Oklejewicz (2006) and Zając and Zając (2019)

*Rubus pedemontanus* Pinkw. = **Rubus nigricans** Danthoine

***Rubus pericrispatus*** Holub & Trávn. (jeżyna kędzierzawa) – the first record of this species from Poland was published by Trávníček and Zázvorka (2005), and its distribution was supplemented by Zając and Zając (2019)

***Rubus perperus*** H.E. Weber (jeżyna różowokwiatowa) – the occurrence of this species in Poland was noted by Oklejewicz in the Carpathians Foothils (FF72 – Grodzisko near Stryżów, FF70 – Gorzejowa Podjaworze, EG09 – Cieklinka Mt., DF91 – Górki), and its distribution map was published by Zając and Zając (2019)

***Rubus perrobustus*** Holub (jeżyna mocna)

***Rubus pfuhlianus*** Sprib. (jeżyna Pfuhla)

***Rubus plicatus*** Weihe & Nees (jeżyna fałdowana)

*Rubus polonicus* Weston – this taxon was lectotypified by Van De Beek (2016), and this name was treated as having priority over *R. nessensis* (Van De Beek 2016; POWO 2025). However, there is no certain evidence that this taxon is fully conspecific with *R. nessensis*. At the same time, it is not yet clear whether the name *R. polonicus* refers only to a local biotype or whether it is sufficiently broadly distributed to be considered a species. At present, the name *R. nessensis*, which is used in the current literature for this widely distributed species, is retained

***Rubus portae-moravicae*** Holub & Trávn. (jeżyna morawska) – the first record from Poland was published by Trávníček and Zázvorka (2005)

***Rubus posnaniensis*** Sprib. (jeżyna poznańska)

***Rubus praecox*** agg. (jeżyna długopręcikowa) – this name is used to designate a group of several taxa from Central Europe, none of which is apparently taxonomically identical to the type material of the name *R. praecox* Bertol. Therefore, this name should be used only for the entire group (*R. praecox* agg.). Similarly, it is not yet clear whether the name *R. procerus* Boulay, currently also used in some literature (Hassler and Muer 2024) for this group, refers to the plants recorded in Poland. The problem requires taxonomic and nomenclatural study

***Rubus prissanicus*** Kosiński, Maliński & Ziel. (jeżyna pyrzycka) – detailed morphological characters and taxonomy of this species, newly described from Poland, were published by Kosiński et al. (2018)

*Rubus × pseudidaeus* (Weihe) Lej. = **Rubus × idaeoides** Ruthe

*Rubus pyramidalis* Kaltenb. = **Rubus umbrosus** (Weihe & Nees) Arrh.

***Rubus radula*** Weihe (jeżyna szorstka)

***Rubus rudis*** Weihe (jeżyna szczeciniasta)

***Rubus salisburgensis*** Focke ex Caflisch (jeżyna salzburska)

***Rubus saxatilis*** L. (malina kamionka)

***Rubus scaber*** Weihe (jeżyna zadzierzysta)

***Rubus schleicheri*** Weihe ex Tratt. (jeżyna Schleichera)

*Rubus schnedleri* H.E. Weber = **Rubus atrovirens** P.J. Müll.

***Rubus sciocharis*** (Sudre) W.C.R. Watson (jeżyna cienista)

***Rubus scissus*** W.C.R. Watson (jeżyna rozcięta)

***Rubus seebergensis*** Pfuhl ex Sprib. (jeżyna mosińska)

***Rubus senticosus*** Köhler ex Weihe (jeżyna górska)

***Rubus siemianicensis*** Sprib. (jeżyna siemianicka)

***Rubus silesiacus*** Weihe (jeżyna śląska)

***Rubus sprengelii*** Weihe (jeżyna Sprengela)

***Rubus spribillei*** (Pfuhl ex Sprib.) Kulesza (jeżyna Spribillego)

***Rubus subbeskidensis*** Wolanin, M. Nobis & Oklej. (jeżyna podbeskidzka) – detailed morphological characters and taxonomy of this species, newly described from Poland, were published by Wolanin et al. (2025) under the name *R. magurensis*

***Rubus subgothicus*** Sprib. (jeżyna nibygocka) – the first record from Poland was published by Trávníček et al. (2021)

***Rubus sulcatus*** Vest (jeżyna bruzdowana)

***Rubus tabanimontanus*** Figert (jeżyna fioletowopędowa)

***Rubus umbrosus*** (Weihe & Nees) Arrh. (jeżyna zacieniona)

***Rubus velutinus*** Vest ex Tratt^.^ (jeżyna aksamitna) – the first record from Poland was published by Király et al. (2017)

***Rubus wahlbergii*** Arrh. (jeżyna Wahlberga)

***Rubus wimmerianus*** (Sprib. ex Sudre) Sprib. (jeżyna Wimmera)

*Rubus xanthocarpus Bureau & Franch. (jeżyna żółtoowockowa)

***Rubus zielinskii*** Wolanin & M.N. Wolanin (jeżyna Zielińskiego) – detailed morphological characters and taxonomy of this species, newly described from Poland, were published by Wolanin et al. (2016)

### *Rubus subbeskidensis* Wolanin, M. Nobis & Oklej., a new name for *R. magurensis* Wolanin, M. Nobis & Oklej.

Wolanin et al. (2025) described *Rubus magurensis* (*Rubus* ser. *Micantes*), a new species from southern Poland. However, they overlooked that the epithet ‘*magurensis*’ had already been used by Nyárády (1956) for a local biotype from Romania, which is known to date from a single locality on Magura Ocnei Mountain (Kurtto et al. 2010; POWO 2025). Thus, a new name for this taxon recently described from Poland needs to be proposed.

#### Rubus subbeskidensis

Taxon classificationPlantaeRosalesRosaceae

Wolanin, M. Nobis & Oklej. nom. nov.

85E9EE62-7E30-5A14-B760-50846506B28C

urn:lsid:ipni.org:names:77383764-1

##### Replaced synonym.

*Rubus magurensis* Wolanin, M. Nobis & Oklej., Forests 16(8): 1286. 2025, nom. illeg. (non *Rubus magurensis* Nyár., Fl. Republ. Popul. Române 4: 908. 1956).

##### Etymology.

The species name *‘subbeskidensis*’ originates from the region where this species occurs, the northern Polish part of the Carpathians.

##### Type.

Poland • Województwo podkarpackie, 1 km na N od Świątkowej Wielkiej, 49°32'15.5"N, 21°26'08.0"E, przy leśnej drodze, [Podkarpackie voivodship, 1 km N of Świątkowa Wielka, 49°32'15.5"N, 21°26'08.0"E, near a forested road], 22 June 2015, M. Wolanin & K. Oklejewicz s.n. (holotype: inflorescence KRA00640849a and primocane leaves KRA00640849b).

It is worth noting that *R. magurensis*, described by Nyárády from Romania, is a member of the series *Sylvatici*, and it has a number of features that readily distinguish it from the species described from Poland, including hairy primocanes without stalked glands and gland-tipped acicles, stronger, more numerous and straighter primocane prickles, longer petiolules and narrower obovate-lanceolate petals (Nyárády 1956; Wolanin et al. 2025).

### Phytogeographical analysis of the micro-hotspots of bramble species richness in Poland

Currently, the genus *Rubus* is represented by 113 taxa in Poland. This number includes 106 native species and seven alien taxa established in the local flora. Two species, *R. × idaeoides* and *R. × areschoughii*, are of hybrid origin. Currently, 12 species are known exclusively from Poland and can be treated as local endemics.

**Figure 1.**
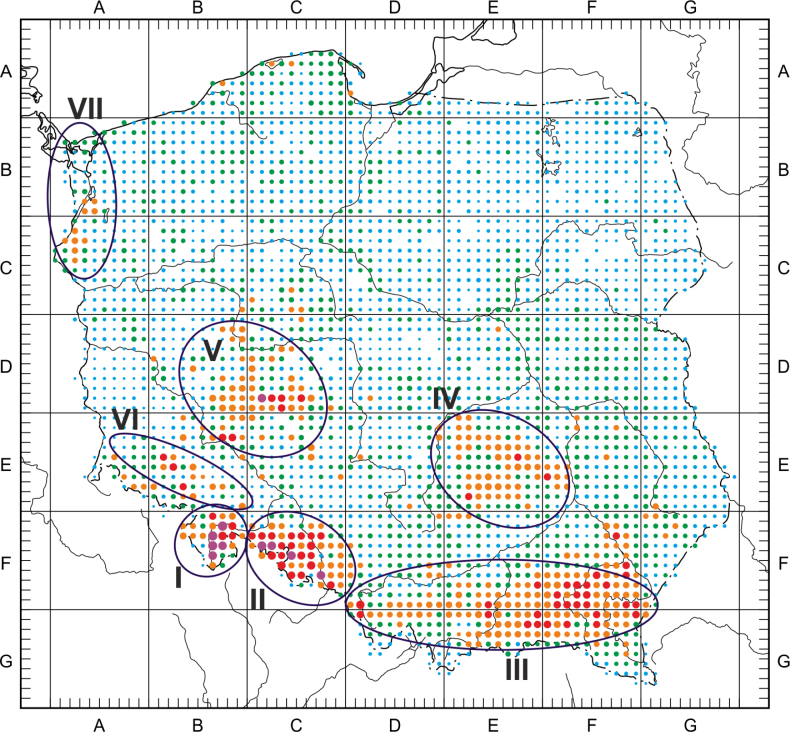
A map of the species richness of brambles in Poland in the ATPOL grid of 10 × 10 km squares, based on the distribution of 113 species. The diameter and colour of points on the map denote the number of species in particular cartogram units: purple – 30–37 species, red – 20–29 species, orange – 10–19 species, green – 5–9 species and blue – 1–4 species. Names of the micro-hotspots marked by ellipses are: I) Kotlina Kłodzka Basin, II) Moravian Gate, III) forelands of the northern Carpathians, IV) Wyżyna Małopolska Upland, V) Wielkopolska Lowland, VI) north-eastern foreland of the Sudetes Mts and VII) Lower Oder River valley.

The analysis of the collective distribution of the 113 species shows that there are 75 cartogram units in which the number of species recorded is higher than 20. The highest concentration of *Rubus* species is observed in south-western Poland, where more than 70% of the Polish brambles occur. However, based on the analysis of the species-richness map (Fig. 1, Table 1), at least seven regions with the highest species concentration can be distinguished as local micro-hotspots of brambles in Poland. These include: I) Kotlina Kłodzka Basin (including Bardo Mts, Bystrzyckie Mts, Kotlina Kłodzka Basin, Złote Mts, Masyw Śnieżnika Mts and the neighbouring eastern foreland of the Sudetes in the Nysa Kłodzka River valley), II) Moravian Gate (mainly Opawskie Mts, Głubczycki Plateau, Kotlina Raciborska Basin and the southern part of the Silesian Lowland), III) forelands of the northern Carpathians (together with the neighbouring southern part of Kotlina Sandomierska Basin), IV) Wyżyna Małopolska Upland (mainly Świętokrzyskie (Holy Cross) Mts and neighbouring areas), V) Wielkopolska Lowland, VI) north-eastern foreland of the Sudetes Mts and VII) Lower Oder River valley.

**Table 1.** Characteristics of the bramble micro-hotspots in Poland. Abbreviations: m-h I – Kotlina Kłodzka Basin; m-h II – Moravian Gate; m-h III – forelands of the northern Carpathians; m-h IV – Wyżyna Małopolska Upland; m-h V – Wielkopolska Lowland; m-h VI – north-eastern foreland of the Sudetes Mts; and m-h VII – Lower Oder River valley.

**Character**	**m-h I**	**m-h II**	**m-h III**	**m-h IV**	**m-h V**	**m-h VI**	**m-h VII**
Number of units (10 × 10 km) per area	28	58	230	120	120	55	80
Number of species in the region	37	33	28	20	30	23	16
Name of the richest unit (10 × 10 km) in species of *Rubus*	BF17	CF31	DF91	EE47, EE82, FE60	CD81	BE52	AC22
Number of units with over 30 species	5	4	–	–	1	–	–
Number of units with 20–29 species	4	19	29	3	6	3	–
Number of units with 10–19 species	11	22	117	49	52	12	10
Number of endemic/regional species in micro-hotspot	4	5	3	1	4	0	2

The highest species richness of brambles in Poland was recorded in the Kotlina Kłodzka Basin micro-hotspot, with the highest number – 37 species – observed in square BF17. Although it is not a particularly large area, covering 28 cartogram units of 10 × 10 km, the number of species in 20 of these units is higher than 10, and in six of the units more than 29 species occur. In three bramble micro-hotspots, more than 30 species occur, while in six of them, more than 17 species occur in 129 cartogram units. With the exception of the north-eastern foreland of the Sudetes Mts, the occurrence of at least one endemic bramble species was confirmed in all other micro-hotspots, with the highest number (4–5 species) noted in the Kotlina Kłodzka Basin, Moravian Gate and Wielkopolska Lowland. The highest number of units with more than 10 species per unit is observed in the forelands of the northern Carpathians (146 cartogram units). This is the largest *Rubus* micro-hotspot in Poland, which can even be divided into two sub-micro-hotspots: i) western Carpathian, with the highest species richness observed in the Beskid Śląski Mts; and the much larger ii) eastern Carpathian, including Beskid Niski Mts, Pogórze Jasielskie Foothills, Pogórze Rzeszowskie Foothills, Pogórze Przemyskie Foothills, Pogórze Dynowskie Foothills, Pogórze Ciężkowickie Foothills, Sanocko-Turczyńskie Mts and northern Bieszczady Mts (Fig. 1, Table 1). It is worth noting that although the lowest number of species is observed in the Lower Oder River valley micro-hotspot (with 17 out of 80 units containing more than 10 species), two endemic species occur in the region. It is expected that, after detailed research in the near future, the number of *Rubus* species recorded in this region is likely to increase.

The high diversity of *Rubus* species in the above-listed micro-hotspots undoubtedly results from the variability and mosaic of habitats preferred by individual species, i.e., the presence of disturbed habitats mainly within the range of mesophilous deciduous forests and mixed coniferous-deciduous forests, on moist, fertile or moderately fertile soils. The species richness of *Rubus* clearly decreases in the lower montane zone and in the north-eastern part of the Polish lowlands, along with the diminishing influence of the sub-Atlantic climate (Maliński 2001; Oklejewicz 2006). However, the species composition in particular micro-hotspots confirms that their species arrived from various directions, i.e., from the south, east, west and north. This phenomenon was also confirmed by Zieliński et al. (2004), who studied the south-eastern part of Lower Silesia, an area corresponding to the Moravian Gate micro-hotspot. Zieliński et al. (2004) also stressed that, although southern species could cross not only the Moravian Gate but also the relatively low mountain ranges, the role of the Moravian Gate, especially before its deforestation, in the migration of *Rubus* species to Poland seems to be crucial. Further studies dedicated to the ecology and distribution of *Rubus*, especially in southern and western Poland, are needed to better understand the migration routes of brambles, particularly under the impact of current climate change.

It is also worth noting that each of the aforementioned micro-hotspots includes a number of local biotypes of brambles, most of which are of hybrid origin, thus increasing the total number of genetic entities and making their identification difficult. Moreover, a few more *Rubus* species may potentially be found in Poland, since they grow in neighbouring countries, especially in Germany and Czechia, and their localities are noted or their ranges end near or directly on the Polish border. Examples of such species are: *R. adpressus* Weihe ex H.E. Weber, *R. austromoravicus* Holub, *R. balticus* (Focke) E.H.L. Krause, *R. dethardingii* E.H.L. Krause, *R. geminatus* H.E. Weber, *R. glaucovirens* Maass, *R. haesitans* Martensen & Walsemann, *R. hadracanthos* G. Braun, *R. holandrei* P.J. Müll., *R. integribasis* P.J. Müller ex Boulay, *R. josholubii* H.E. Weber, *R. lobatidens* H.E. Weber & Stohr, *R. leuciscanus* E.H.L. Krause, *R. placidus* H.E. Weber, *R. ranftii* H.E. Weber, *R. stohrii* H.E. Weber & Ranft, *R. vratnensis* Holub, *R. walsemannii* H.E. Weber and *R. wessbergii* A. Pedersen & Walsemann. Thus, there is a clear need for field studies dedicated to finding these taxa in Poland.

### Geographical elements in the *Rubus* flora in Poland

It should be stressed that the distribution of species, especially within such a complicated and taxonomically difficult group as brambles, easily dispersed by animals, mainly birds, always reflects the current state of research. However, a detailed analysis of the distribution patterns of all *Rubus* species in Poland allows the distinction of four main geographical groups of taxa corresponding to the following elements: species reaching their northern, north-eastern, eastern and southern distribution limits in Poland, which are generally correlated with the impact of specific bioclimatic and environmental factors. The following were excluded from the analysis: local endemic species; some species with disjunct distributions, such as *R. atrovirens* or *R. graecensis*; non-native naturalised species, except for *R. armeniacus*; and brambles more or less common in Poland that do not exhibit any geographical correlations, i.e., *R. caesius*, *R. gracilis*, *R. idaeus*, *R. nigricans*, *R. plicatus*, *R. saxatilis*, *R. sulcatus*, *R. nessensis*, *R. × areschoughii* and *R. × idaeoides*.

#### Species with their northern limit in Poland

**Figure 2.**
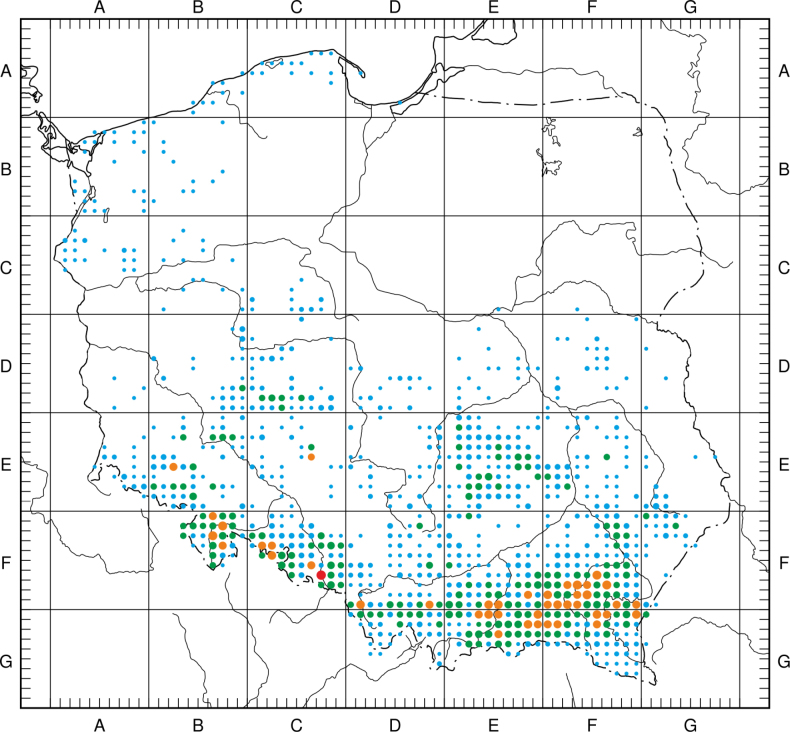
A map of the species concentration of brambles, based on the distribution of 21 species reaching their northern limit in Poland. The diameter and colour of points on the map denote the number of species in particular cartogram units: red – 10 species, orange – 7–9 species, green – 4–6 species and blue – 1–3 species. The map is based on the ATPOL grid of 10 × 10 km squares.

In this group, 21 primarily mountain species, occurring mostly in the southern part of Poland, can be distinguished. The following species belong here: *Rubus apricus*, *R. austroslovacus*, *R. bifrons*, *R. canaliculatus*, *R. constrictus*, *R. crispomarginatus*, *R. divulgatus*, *R. glandulosus*, *R. glivicensis*, *R. guentheri*, *R. henrici-egonis*, *R. kuleszae*, *R. nemoralis*, *R. nemorosus*, *R. orthostachys*, *R. parthenocissus*, *R. pericrispatus*, *R. perrobustus*, *R. sprengelii*, *R. velutinus* and *R. wimmerianus*. The highest concentration of species affiliated with this group is observed in the Western Carpathians and their northern forelands, the Sudetes Mts and their north-eastern foreland, as well as in the Głubczycki Plateau. In over 100 cartogram units, 5 to 8 species per unit occur in these regions. The highest diversity, 10 species per unit, is noted in unit CF67, between Racibórz and Rybnik, whereas 9 species per unit were observed in six units. A slightly lower species concentration is observed in Central Poland, in the Świętokrzyskie (Holy Cross) Mts and Wielkopolska Lowland, where, on average, 5 species per unit were recorded (Fig. 2), whereas not more than one, rarely two species of this group were recorded in northern Poland. It is also worth noting that, apart from *R. glandulosus*, which is the most common mountain species, only *R. idaeus* (one of the most common species in Poland: Zając and Zając 2001; Zieliński 2004) occurs at elevations above 1,000 m (Oklejewicz 2006). Additionally, although some species, such as *R. nemoralis*, *R. nemorosus* or *R. sprengelii*, seem to reach their northern rather than eastern or north-eastern limit in Poland, their localities in Poland occur at the northern limit of their local range; thus, they were included in this species group. The greatest impact on the distribution pattern of species representing this group in Poland seems to be exerted by: I) the mean temperature in the warmest season (summer), which in forelands and lower mountain areas (in southern and central Poland) is slightly more temperate than in the northern part, II) higher precipitation (mean annual precipitation exceeding 600 mm: Lorenc 2005) and III) more fertile and moist soil types.

#### Species with their north-eastern limit in Poland

This group comprises 32 species related mainly to a warmer, sub-Atlantic climate, distributed mainly in the widely understood south-western Poland and spreading via the Moravian Gate. The following species belong here: *Rubus angustipaniculatus*, *R. bavaricus*, *R. bicolor*, *R. bohemo-polonicus*, *R. camptostachys*, *R. capricollensis*, *R. chaerophylloides*, *R. chaerophyllus*, *R. clusii*, *R. divaricatus*, *R. dollnensis*, *R. fabrimontanus*, *R. fasciculatus*, *R. franconicus*, *R. grabowskii*, *R. gracilis*, *R. graecensis*, *R. guttiferus*, *R. koehleri*, *R. macrophyllus*, *R. mollis*, *R. montanus*, *R. portae-moravicae*, *R. posnaniensis*, *R. praecox* agg., *R. radula*, *R. rudis*, *R. salisburgensis*, *R. siemianicensis*, *R. silesiacus*, *R. subgothicus* and *R. tabanimontanus*. It is worth noting that although some species, such as *R. clusii*, *R. montanus* or *R. guttiferus*, seem to reach their northern rather than north-eastern limit in Poland, the Polish localities of these species occur more at the north-eastern general range limit of those species. These taxa were found in Poland fairly recently, and knowledge of their range will be expanded in the near future. Thus, bearing this in mind, they were assigned to this species group.

**Figure 3.**
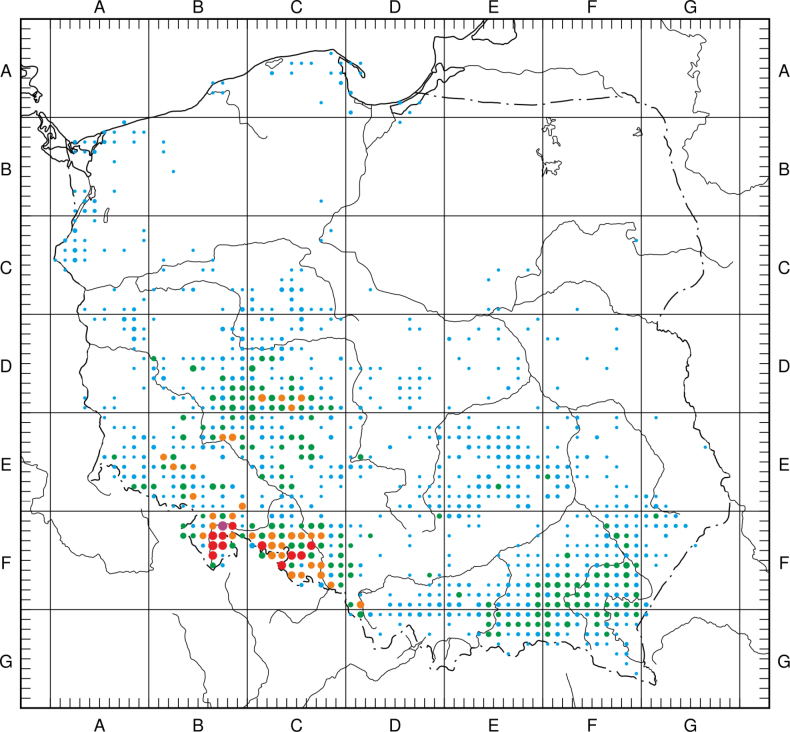
A map of the species concentration of brambles, based on the distribution of 32 species reaching their north-eastern limit in Poland. The diameter and colour of points on the map denote the number of species in particular cartogram units: purple – 17 species, red – 12–16 species, orange – 8–11 species, green – 4–7 species and blue – 1–3 species. The map is based on the ATPOL grid of 10 × 10 km squares.

The highest concentration of species reaching their north-eastern limit in Poland is observed in the south-eastern Sudetes Mts, mainly in the Kotlina Kłodzka Basin, Opava Mts, Głubczycki Plateau, the upper Oder River valley and the Beskid Śląski Mts (Polish Western Carpathians; Fig. 3). The highest species concentration in that area, ranging from 10 to 16 (on average 14) species per cartogram unit, was recorded in 16 units. The highest diversity, 17 species, was observed in unit BF17, between Bardo and Kamieniec Ząbkowicki, in the Sudetes Foreland (Fig. 3). In other regions of south-western Poland, e.g., in the Carpathian Foreland and Sandomierska Basin, due to the impact of the sub-Atlantic climate, 2–6 species (4 on average) representing this geographical element are observed. A slightly higher number of species per unit is noted in the region of Wielkopolska Lowland, with 6, on average, and a maximum of 11 species per unit. Species representing this geographical element rarely extend further to the north-east, beyond the Vistula and Wieprz River valleys (Fig. 3). Although analyses of future distribution changes are beyond the scope of the present study, the observed climate warming may strongly influence the spread and distribution patterns of some species belonging to the analysed group. The observed concentration of thermophilous or sub-Atlantic species in south-western Poland (mainly in the Kotlina Kłodzka Basin and the Moravian Gate) may partly facilitate their further expansion into the interior of the country in the future.

#### Species with their eastern limit in Poland

**Figure 4.**
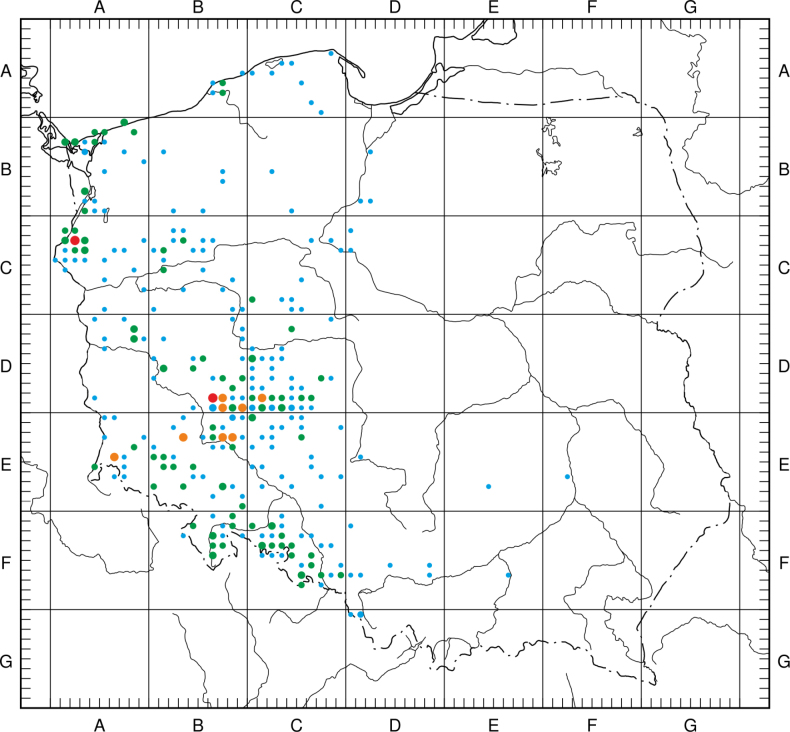
A map of the species concentration of brambles, based on the distribution of 24 species reaching their eastern limit in Poland. The diameter and colour of points on the map denote the number of species in particular cartogram units: red – 5 species, orange – 4 species, green – 3 species and blue – 1–2 species. The map is based on the ATPOL grid of 10 × 10 km squares.

In this group, 24 species distributed mainly in western Poland can be distinguished, which, similarly to the previous group, are also related to the sub-Atlantic, warmer and slightly more humid climate. The following species belong here: *Rubus acanthodes*, *R. armeniacus*, *R. barberi*, *R. chlorothyrsos*, *R. circipanicus*, *R. curvaciculatus*, *R. gothicus*, *R. hercynicus*, *R. hevellicus*, *R. lamprocaulos*, *R. lidforsii*, *R. lignicensis*, *R. lindebergii*, *R. lusaticus*, *R. marssonianus*, *R. maximus*, *R. opacus*, *R. pallidus*, *R. umbrosus*, *R. scaber*, *R. schleicheri*, *R. sciocharis*, *R. senticosus* and *R. wahlbergii*. The highest concentration of species belonging to this group is observed in the south-eastern Sudetes Foreland, Wielkopolska Lowland, Myślibórz Lake District region and Wolin Island (Fig. 4). In those areas, 2–4 species per unit were recorded. The highest number of species (5 per unit) was noted in two squares, AC22 near Lisie Pole village and BD86 near Góra city (SSE from Leszno).

#### Species with their southern limit in Poland

**Figure 5.**
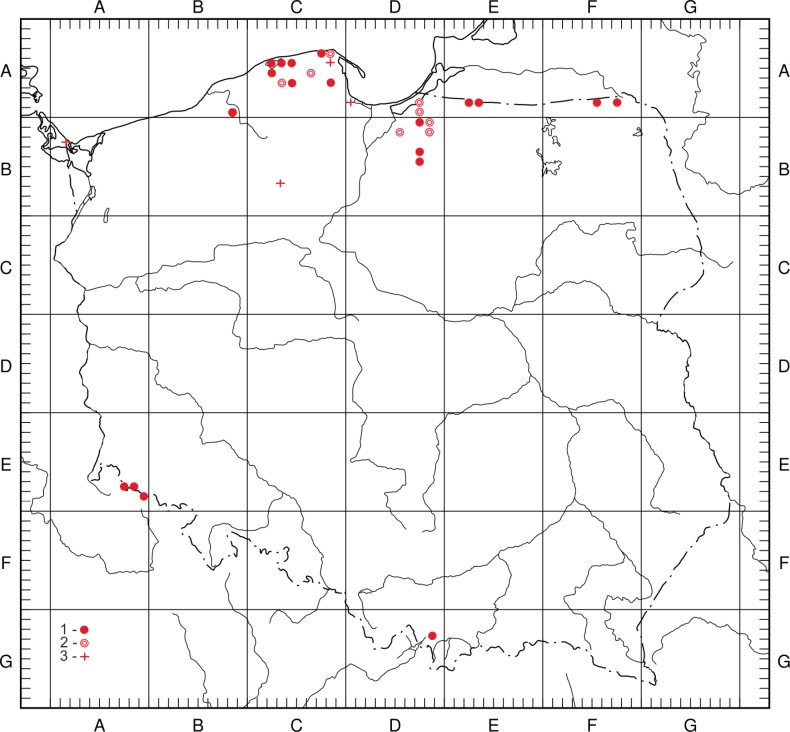
A distribution map of *Rubus chamaemorus* in Poland, a species at its southern limit in the country, in the ATPOL grid of 10 × 10 km squares. Notes: 1 – currently confirmed, 2 – not confirmed, 3 – extinct.

This element is represented only by *Rubus chamaemorus*, distributed mainly in northern Poland with isolated, postglacial relict localities in the Karkonosze Mts and Kotlina Orawsko-Nowotarska Basin (Fig. 5; Koczur 2004; Kruszelnicki et al. 2014). The species is the only protected bramble in Poland, and mainly due to the negative impact of climate change, mostly increasing temperatures resulting in progressive habitat desiccation, its populations have not been currently confirmed in many localities. Therefore, it has been included in the Red Data Book and Red Data List of Plants and is treated as endangered in Poland (category EN; Kruszelnicki et al. 2014; Kaźmierczakowa et al. 2016).

#### Regional (endemic) species

There are 12 species whose distribution is restricted to Poland. Most of these can be considered local endemics; however, it is quite probable that populations of some of these species will be found in Germany or Czechia. The following species belong here: *R. czarnunensis* (probably endemic to Poland), *R. kaznowskii* (probably endemic to Poland), *R. limitaneus* (regional species possibly occurring in Germany), *R. oboranus* (probably endemic to Poland), *R. oklejewiczii* (probably endemic to Poland), *R. ostroviensis* (endemic to Poland), *R. pfuhlianus* (endemic to Poland), *R. prissanicus* (regional species, probably endemic to Poland), *R. seebergensis* (probably endemic to Poland), *R. spribillei* (endemic to Poland), *R. subbeskidensis* (probably endemic to Poland) and *R. zielinskii* (endemic to Poland).

Two species previously treated as endemic to Poland – *R. capitulatus* and *R. lucentifolius* (Zieliński 2004) – are excluded from the list since their occurrence has recently been confirmed in northern Czechia (Pladias 2025).

## Supplementary Material

XML Treatment for Rubus subbeskidensis

## Additional information

### Artificial Intelligence (AI) use

The authors accept full responsibility for the content of the manuscript, including the disclosure of any use of AI.

No AI tools were used in the preparation of this manuscript.

### Author ORCIDs

M. Nobis https://orcid.org/0000-0002-1594-2418

M. Wolanin https://orcid.org/0000-0002-2461-5750

B. Trávníček https://orcid.org/0000-0003-4596-2476

### Data availability

All of the data that support the findings of this study are available in the main text.

## References

[B1] ATPOL (2025) Distribution Atlas of Vascular Pants in Poland – database. Available in the Institute of Botany Jagiellonian University in Krakow. [accessed 12 December 2025].

[B2] Gapińska M, Kosiński P (2016) *Rubus* flora of the Durowo Forest District (northern Wielkopolska, Poland). Steciana 20(4): 239–249. 10.12657/steciana.020.025

[B3] Hassler M, Muer T (2024) Flora Germanica: alle Farn- und Blütenpflanzen Deutschlands in Text und Bild. Band 3, Verlag Regionalkultur, Uberstadt-Weiher, 1–823.

[B4] Jaźwa M, Stadnicka-Futoma A (2017) Flora roślin naczyniowych Podgórza Rzeszowskiego. Polska Akademia Nauk, Komitet Biologii Organizmalnej, Instytut Botaniki Uniwersytetu Jagiellońskiego, 1–453.

[B5] Kaźmierczakowa R, Bloch-Orłowska J, Celka Z, Cwener A, Dajdok Z, Michalska-Hejduk D, Pawlikowski P, Szczęśniak E, Ziarnek K (2016) Polska czerwona lista paprotników i roślin kwiatowych. Instytut Ochrony Przyrody PAN, Kraków, 1–44.

[B6] Király G, Sochor M, Trávníček B (2017) Reopening an old chapter: a revised taxonomic and evolutionary concept of the *Rubus montanus* group. Preslia 89: 309–331. 10.23855/preslia.2017.309

[B7] Koczur A (2004) Newly discovered relic population of *Rubus chamaemorus* L. in the Western Carpathians. Acta Societatis Botanicorum Poloniae 73(2): 129–133. 10.5586/asbp.2004.018

[B8] Kollmann J, Steinger T, Roy BA (2000) Evidence of sexuality in European *Rubus* (Rosaceae) species based on AFLP and allozyme analysis. American Journal of Botany 87: 1592–1598. 10.2307/2656735 11080109

[B9] Kosiński P (2006) Current distribution of the recently described bramble species, *Rubus guttiferus* (Rosaceae), in Poland. Dendrobiology 56: 45–50.

[B10] Kosiński P (2010) The genus *Rubus* in the Bardo Mts (Central Sudetes). Dendrobiology 63: 77–98.

[B11] Kosiński P, Oklejewicz K (2006) *Rubus parthenocissus* (Rosaceae) in Poland. Dendrobiology 55: 33–38.

[B12] Kosiński P, Zieliński J (2013) *Rubus maximus* (Rosaceae) found also in Poland. Botanika-Steciana 17: 33–37.

[B13] Kosiński P, Czarna A, Malinski T (2014) *Rubus occidentalis* (Rosaceae) – A new naturalized raspberry species in the Polish flora. Dendrobiology 71: 159–165. 10.12657/denbio.071.016

[B14] Kosiński P, Maliński T, Sliwinska E, Zieliński J (2018) *Rubus prissanicus* (Rosaceae), a new bramble species from North West Poland. Phytotaxa 344: 239–247. 10.11646/phytotaxa.344.3.4

[B15] Kosiński P, Maliński T, Nobis M, Rojek-Jelonek M, Tomaszewski D, Dering M, Zieliński J (2021) *Rubus kaznowskii* (Rosaceae), a new bramble species from south-central Poland. PhytoKeys 185: 27–41. 10.3897/phytokeys.185.71193 PMC8604876 34819779

[B16] Kruszelnicki J, Fabiszewski J, Koczur A (2014) *Rubus chamaemorus* L. Malina moroszka. In: Kaźmierczakowa R, Zarzycki K, Mirek Z (Eds) Polska czerwona księga roślin. Paprotniki i rośliny kwiatowe. Instytut Ochrony Przyrody, Polska Akademia Nauk, Kraków, 258–259.

[B17] Kurtto A, Weber HE, Lampinen R, Sennikov AN [Eds] (2010) Atlas Florae Europaeae: Distribution of Vascular Plants in Europe; 15. Rosaceae (*Rubus*). The Committee for Mapping the Flora of Europe & Societas Biologica Fennica Vanamo: Helsinki, Finland, 1–362.

[B18] Lorenc H [Ed.] (2005) Atlas klimatu Polski, IMGW, Warszawa, 1–116.

[B19] Maliński T (2001) Rodzaj *Rubus* L. w południowej Wielkopolsce. Rocznik Dendrologiczny 49: 13–95.

[B20] Maliński T, Zieliński J, Kosiński P (2014) *Rubus limitaneus* (series Mucronati, subgenus *Rubus*, Rosaceae) – A species new to science from NW Poland. Dendrobiology 72: 57–64. 10.12657/denbio.072.005

[B21] Maliński T, Zieliński J, Kosiński P (2015) *Rubus lindebergii* (Rosaceae) – New species for the flora of Poland. Dendrobiology 74: 143–147. 10.12657/denbio.074.014

[B22] Mirek Z, Piękoś-Mirkowa H, Zając A, Zając M (2020) Vascular Plants of Poland: An Annotated Checklist. W. Szafer Institute of Botany, Polish Academy of Sciences, Kraków, 1–526.

[B23] Nobis A (2008) Rośliny naczyniowe wschodniej części Kotliny Sandomierskiej. Zeszyty Naukowe Uniwersytetu Jagiellońskiego, Prace Botaniczne 42: 1–341.

[B24] Nobis M (2007) Rośliny naczyniowe zachodniej części Przedgórza Iłżeckiego (Wyżyna Małopolska). Zeszyty Naukowe Uniwersytetu Jagiellońskiego, Prace Botaniczne 40: 1–458.

[B25] Nyárády EI (1956) *Rubus*. In: Săvulescu T (Ed.) Flora Republicii Populare Române (Vol. IV). Academiei Republicii Populare Române Press, Bucharest, 276–580.

[B26] Nykiel M, Wolanin M, Oklejewicz K (2014) *Rubus divaricatus* (Rosaceae) na Płaskowyżu Kolbuszowskim: nowa wschodnia granica zasięgu. Acta Botanica Silesiaca 10: 199–206.

[B27] Oklejewicz K (2006) Distribution patterns of *Rubus* species (Rosaceae) in the eastern part of the Polish Carpathians. Polish Botanical Studies 21: 1–98.

[B28] Oklejewicz K, Chwastek E (2017) Nowe stanowiska *Rubus clusii* (Rosaceae) w Karpatach Polskich. Fragmenta Floristica et Geobotanica Polonica 24(2): 507–508.

[B29] Oklejewicz K, Trávníček B, Wolanin M (2013) New localities of *Rubus clusii* (Rosaceae) seriously expanding it range towards the East. Dendrobiology 70: 93–98. 10.12657/denbio.070.010

[B30] Pierścińska A (2014) Rośliny naczyniowe wschodniej części Niecki Połanieckiej (Wyżyna Małopolska) i przyległej części Niziny Nadwiślańskiej (Kotlina Sandomierska). Zeszyty Naukowe Uniwersytetu Jagiellońskiego, Prace Botaniczne 45: 1–354.

[B31] Piwowarczyk R (2008) Rośliny naczyniowe wschodniej części Przedgórza Iłżeckiego (Wyżyna Małopolska). Zeszyty Naukowe Uniwersytetu Jagiellońskiego, Prace Botaniczne 43: 1–344.

[B32] Pladias (2025) Pladias Database of the Czech Flora and Vegetation. https://pladias.cz/en/ [accessed 12 December 2025]

[B33] POWO (2025) Plants of the World Online. Facilitated by the Royal Botanic Gardens, Kew. https://powo.science.kew.org/ [accessed 9 August 2025]

[B34] Sochor M, Lepší M, Lepší P, Valebil J, Király G, Trávníček B (2025) Taxonomy and nomenclature of *Rubus* ser. *Glandulosi* (Rosaceae) across its Eurasian range: Revised concepts and new approaches. Taxon 74: 1106–1152. 10.1002/tax.13380

[B35] Thiers B (2025) Index Herbariorum: A Global Directory of Pub-lic Herbaria and Associated Staff. New York Botanical Garden’s Virtual Herbarium. http://sweetgum.nybg.org/science/ih/ [accessed 10 November 2025]

[B36] Trávníček B, Zázvorka J (2005) Taxonomy of *Rubus* ser. Discolores in the Czech Republic and adjacent regions. Preslia 77: 1–88.

[B37] Trávníček B, Oklejewicz K, Zieliński J (2005) *Rubus ambrosius* (*Rubus* subsect. *Rubus*, Rosaceae), a new bramble species from the eastern part of Central Europe. Folia Geobotanica 40: 421–434. 10.1007/BF02804289

[B38] Trávníček B, Sochor M, Kosiński P, Király G (2021) Taxonomy of the *Rubus gothicus* group in south-eastern central Europe. Preslia 93: 321–340. 10.23855/preslia.2021.321

[B39] Van De Beek A (2016) Validations of the *Rubus* taxa in Tournefort’s *Institutiones* and their *Corollarium* in later literature. Adansonia 38(1): 35–53. 10.5252/a2016n1a4

[B40] Wolanin M (2013) Locality of *Rubus chaerophylloides* (Rosaceae) in south-eastern Poland. Fragmenta Floristica et Geobotanica Polonica 20(1): 140–142.

[B41] Wolanin M, Musiał K, Nobis M (2020) *Rubus oklejewiczii* (Rosaceae), a new bramble species from Central Europe (Poland: Carpathians). Phytotaxa 438: 189–198. 10.11646/phytotaxa.438.3.3

[B42] Wolanin M, Musiał K, Nobis M (2025) *Rubus magurensis* (Rosaceae): A New Bramble Species from the Northern Carpathians (Poland). Forests 16(8): 1286. 10.3390/f16081286

[B43] Wolanin MM, Wolanin MN, Musiał K, Kania I, Oklejewicz K (2016) *Rubus zielinskii* (Rosaceae), a new species from Poland. Phytotaxa 273: 183–190. 10.11646/phytotaxa.273.3.5

[B44] Zając A (1978) Założenia metodyczne “Atlasu rozmieszczenia roślin naczyniowych w Polsce”. Wiadomości Botaniczne 22(3): 145–155.

[B45] Zając A, Zając M [Eds] (2001) Atlas rozmieszczenia roślin naczyniowych w Polsce. Nakładem Pracowni Komputerowej Instytutu Botaniki Uniwersytetu Jagiellońskiego, Kraków, 1–714.

[B46] Zając A, Zając M [Eds] (2019) Atlas rozmieszczenia roślin naczyniowych w Polsce. Dodatek. Instytut Botaniki Uniwersytetu Jagiellońskiego, Kraków, 1–319.

[B47] Zając M, Zając A (2009) Elementy geograficzne rodzimej flory Polski. Nakładem Pracowni Komputerowej Instytutu Botaniki Uniwersytetu Jagiellońskiego, Kraków, 1–93.

[B48] Zieliński J (2004) The genus *Rubus* (Rosaceae) in Poland. Polish Botanical Studies 16: 1–300.

[B49] Zieliński J, Trávníček B (2004) *Rubus bohemo-polonicus* (Rosaceae) – a new species of bramble from the Czech Republic and Poland. Acta Societatis Botanicorum Poloniae 73(4): 311–314. 10.5586/asbp.2004.040

[B50] Zieliński J, Kosiński P, Tomaszewski D (2004a) *Rubus lucentifolius* (Rosaceae), a new species of bramble from Poland. Polish Botanical Journal 49(1): 5–9.

[B51] Zieliński J, Kosiński P, Tomaszewski D (2004b) The genus *Rubus* (Rosaceae) in southeastern Lower Silesia (Poland). Polish Botanical Journal 49(2): 161–180.

